# Autophagy: the pathogenic agent in muscle damage

**DOI:** 10.1186/1471-2474-14-S2-O1

**Published:** 2013-05-29

**Authors:** Nina Raben

**Affiliations:** 1 Laboratory of Muscle Stem Cells and Gene Regulation, National Institute of Arthritis and Musculoskeletal and Skin Diseases, National Institutes of Health, Bethesda, Maryland, USA

## 

Recently autophagy has attracted considerable attention because of its role in a wide variety of diseases including neurodegenerative disorders, cancer, myopathies, and lysosomal storage diseases. Autophagy is a “self-eating” process that brings proteins and damaged organelles enclosed in double-membrane autophagosomes to lysosomes for digestion and recycling. Functional lysosomes are essential for the completion of autophagy-initiated degradation and recycling of cellular components. In the fatal lysosomal glycogen storage disorder, Pompe disease, dysfunctional autophagy and massive accumulation of autophagic debris in myofibers greatly contribute to the cellular damage and interfere with the efficacy of enzyme replacement therapy (ERT). Analysis of single muscle fibers from patients with Pompe disease confirmed that the autophagic inclusions are prominent in humans as well. Autophagic buildup persists after years of treatment and may well be the reason for disappointing clinical response. Genetic suppression of autophagy in a mouse model of Pompe disease reduced the lysosomal glycogen load and allowed for fully effective ERT in murine Pompe disease. Our group is currently exploring a new therapeutic approach to Pompe disease; this new approach involves manipulation of transcription factor EB (TFEB), which has been shown to promote lysosomal-autophagosomal fusion and biogenesis. The appeal for Pompe disease is that unlike the current therapy, modulation of TFEB holds promise to rid muscle cells of both excessive glycogen burden and accumulation of autophagic debris.

